# Program Directors’ Opinions About Otolaryngology Resident Teaching Medical School Anatomy

**DOI:** 10.7759/cureus.10999

**Published:** 2020-10-17

**Authors:** Tyler Wanstreet, Sarah Callaham, Daniel O'Brien, Michele M Carr

**Affiliations:** 1 Medicine, West Virginia University School of Medicine, Morgantown, USA; 2 Otolaryngology, West Virginia University, Morgantown, USA; 3 Otolaryngology, Jacobs School of Medicine and Biomedical Sciences, University at Buffalo, Buffalo, USA

**Keywords:** teaching anatomy, medical education, residents as teachers, medical students, program directors, otolaryngology education

## Abstract

Purpose

To evaluate whether otolaryngology residency program directors (PDs) provide residents to teach pre-clinical medical students anatomy and to outline their perceptions of this practice.

Methods

An anonymous online survey was sent to active U.S. otolaryngology residency PDs in 2019, assessing each program’s involvement in teaching medical student anatomy.

Results

Forty-five of 121 (37.1%) of surveyed otolaryngology PDs responded. Sixteen of the 44 (36.4%) residency programs that were associated with a medical school provided residents to teach anatomy (“Teaching Programs”). The 29 (64.4%) remaining programs did not provide residents (“Non-teaching Programs”). No significant differences were found between Teaching and Non-teaching Programs (P<0.05) for the size of the program, the presence of fellowships, the size of medical school, whether residents had won teaching awards, or the number of otolaryngology residency applicants from that school. In general, all PDs responded positively about residents teaching medical school anatomy. Non-teaching Programs primarily cited not being approached by the medical school as a reason for not providing residents to teach.

Conclusion

The majority of respondent otolaryngology PDs have a positive view of residents teaching medical students but few do it. Otolaryngology departments will need to take the lead on developing opportunities to put students and residents together for anatomy education.

## Introduction

Involving residents in the education of pre-clinical medical students has become a common practice in many medical schools. Residents teaching anatomy has been shown to increase the understanding and confidence that students have in the subject and to amplify student interest in the residents’ particular specialties [1-8]. Interacting with residents also allows students to gain positive role models and gives them opportunities to seek one-on-one advice about medical education and learn about residency programs and resident lifestyles early in their medical school career [9].

This practice has been shown to increase the confidence residents have in their teaching abilities and provides residents the opportunity to review anatomy in a low-stress environment [1,5,8,10]. These teaching skills may carry over into their future medical practice and improve the residents’ ability to teach their patients about their disease pathology and prognosis, surgical procedures, and the benefits and risks of medications.

Although these mutual benefits are known, there is little data concerning the prevalence of otolaryngology resident involvement in teaching head and neck anatomy to pre-clinical medical students. The number of residents invited to teach, the amount of time allocated to teach, and the roles they play while teaching are also not well-investigated. In this study, we surveyed otolaryngology residency program directors (PDs) to determine if their residents were teaching medical school anatomy, whether the PD perceived benefits from it, and how the program managed the logistics of resident involvement.

## Materials and methods

This study was approved by the local university institutional review board. PDs of all United States active allopathic and osteopathic otolaryngology residency programs were recruited via secure, confidential electronic mail messages in Spring 2019. Email addresses were obtained through online searches and the Accreditation Council for Graduate Medical Education (ACGME) website (www.acgme.org). Each electronic mail message contained a link to an anonymous online survey powered by REDCap (Vanderbilt University, Nashville, Tenn.) as well as an explanation of the study. The survey was designed to assess each residency program’s involvement in teaching medical student anatomy at their institution. Participants who did not complete the survey within two weeks were sent an automated email reminder. Reminders were then sent at intervals but did not exceed five attempts. Aggregate data was entered on a password-protected spreadsheet and summarized using descriptive statistics and compared using nonparametric methods with Statistical Package for the Social Sciences (SPSS) 25.0 (2017, IBM Corp., Armonk, NY). The chi-square test was used for binary data and the Kruskal-Wallis test for multiple groups, with level of significance p<0.05.

## Results

A total of 121 otolaryngology residency programs were initially contacted. Survey responses were received from 45 of the 121 programs (37.1%). Forty-three out of 45 of the surveys (95.6%) were completed by otolaryngology residency PDs, one by an assistant or associate PD, and the last by an attending surgeon responsible for teaching residents. The mean number of residents was 13.6 per program. The mean number of fellows was 1.52 per program. Twenty-one of the 45 programs (46.7%) did not have a fellow. One program was not associated with a medical school. Additional data regarding the size of the residency programs and the institutions’ associated graduating medical school classes can be found in Table 1.

**Table 1:** Demographic Information About Respondent Programs No significant differences; p>0.05

		Teaching Program n (%)	Non-Teaching Program n (%)	Total n (%)
Size of Residency Program (# of Residents)	<10	7 (43.8)	11 (37.9)	18 (40.0)
	11 – 20	9 (56.3)	14 (48.3)	23 (51.1)
	21+	0	4 (13.8)	4 (8.9)
	Total	16 (100.0)	29 (100.0)	45 (100.0)
Size of Fellowship Program (# of Fellows)	0 – 3	16 (100.0)	22 (78.6)	38 (86.4)
	4 – 6	0	3 (10.7)	3 (6.8)
	7 – 9	0	3 (10.7)	3 (6.8)
	Total	16 (100.0)	28 (100.0)	44 (100.0)
Size of Graduating Medical School Class (# of Students)	<100	0	4 (13.8)	4 (8.9)
	101 – 150	7 (43.8)	10 (34.5)	17 (37.8)
	151 – 200	7 (43.8)	7 (24.1)	14 (31.1)
	>201	2 (12.5)	7 (24.1)	9 (20.0)
	N/A	0	1 (3.4)	1 (2.2)
	Total	16 (100.0)	29 (100.0)	45 (100.0)

A total of 16 of 44 programs associated with a medical school (36.4%) indicated at least one resident or fellow was currently involved in teaching anatomy to medical students. These were deemed “Teaching Programs.” Fifteen (93.8%) of the Teaching Programs utilized only residents while one (6.7%) program involved both residents and fellows. Residents in 13 of the Teaching Programs (81.3%) assisted in the anatomy lab. Two programs (12.5%) also had their residents teach in a lecture setting. The remaining three programs (18.8%) had their residents teach anatomy in another, unspecified capacity. The mean number of residents provided to instruct per Teaching Program was 3.56. Over the course of an academic year, the majority of residents devoted two days or less to teaching anatomy (range was between <1/2 day to > 3 days). For 10 of the 16 programs (62.5%), the residents’ clinical responsibilities were covered by fellow residents during their teaching time.

The remaining 29 of 44 programs associated with a medical school (65.9%) who did not provide residents to teach anatomy comprise the Non-Teaching Program group. While they do not currently participate, 17 out of 29 Non-Teaching PDs (58.6%) felt utilizing residents to teach anatomy was a good idea. Twenty-seven of the Non-Teaching PDs provided responses to why their residents were not involved in teaching medical school anatomy; respondents were able to select multiple reasons. Thirteen respondents (48.1%) cited that their program had not been approached by the medical school to provide residents for this purpose. Ten (37.0%) noted that the clinical demands on the residents precluded their availability to participate in teaching. Six (22.2%) indicated that too few residents were enrolled in their program to provide this service. Only two PDs (7.4%) stated previous attempts at providing residents had been made, but it was too problematic. No respondent indicated a lack of potential benefits of providing residents as a reason.

Forty respondents indicated whether or not they valued residents teaching medical school anatomy. All 15 Teaching Programs (100.0%) who submitted an answer to this question answered yes, there is value to the practice. Seventeen of the 25 Non-Teaching Programs (68.0%) who answered the question selected yes while the remaining eight (32.0%) selected no. In total, 80.0% of the respondents found value in residents teaching anatomy. Each PD was then asked to assess the specific benefits to the residency program, residents, and medical students. Respondents were able to select multiple benefits. More Teaching Program respondents agreed with each assessed benefit compared to Non-Teaching Program respondents, but this did not reach statistical significance (P>0.05). The results are summarized in Table 2.

**Table 2:** Benefits of Providing Residents to Teach Anatomy No significant differences; p>0.05

		Teaching Program n (%)	Non-Teaching Program n (%)	Total n (%)
Benefits to Residency Programs	Program’s Visibility to Medical Students	14 (87.5)	15 (51.7)	29 (64.4)
	Program’s Reputation in Medical School	10 (62.5)	11 (37.9)	21 (46.7)
	Recruit Potential Future Residents	12 (75.0)	11 (37.9)	23 (51.1)
	Total	16 (100.0)	29 (100.0)	45 (100.0)
Benefits to Residents	Improve Anatomy Knowledge	10 (62.5)	13 (44.8)	23 (51.1)
	Chance to Practice Teaching	14 (87.5)	15 (51.7)	29 (64.4)
	Break from Clinical Duties	3 (18.8)	2 (6.9)	5 (8.9)
	Positive Feedback from Students	7 (43.8)	7 (24.1)	14 (31.1)
	Total	16 (100.0)	29 (100.0)	45 (100.0)
Benefits to Medical Students	Knowledgeable Teacher for Head & Neck Anatomy	10 (62.5)	11 (37.9)	21 (46.7)
	Otolaryngology Resident as Role Model	11 (68.8)	12 (41.3)	23 (51.1)
	Aware of Otolaryngology as Career Choice	10 (62.5)	10 (34.5)	20 (44.4)
	Additional Teaching Staff to Answer Questions	9 (56.3)	7 (24.1)	16 (35.6)
	Total	16 (100.0)	29 (100.0)	45 (100.0)
Resident Teaching Awards Received by Resident in Their Program in Last 5 Years	Received	5 (33.3)	12 (46.2)	17 (41.5)
	Did Not Receive	7 (46.7)	12 (46.2)	19 (46.3)
	No Awards Available	3 (20.0)	2 (7.7)	5 (12.2)
	Total	15 (100.0)	26 (100.0)	41 (100.0)
Medical Students Applying to Otolaryngology Residencies This Year (# of Medical Students)	0 – 2	4 (25.0)	11 (39.3)	15 (34.1)
	3 – 5	9 (56.3)	9 (32.1)	18 (40.9)
	5 – 7	3 (18.8)	8 (28.6)	11 (25.0)
	Total	16 (100.0)	28 (100.0)	44 (100.0)

Non-parametric analyses were performed to assess if any demographic differences influenced whether or not a program’s residents participated in anatomy teaching. Analyses based upon the size of the residency program, if a residency program contained fellows or not, if teaching awards were received by residents or not, and the number of medical students from their institution who applied to otolaryngology residency programs (number of applicants) were assessed, and no significant differences were identified.

## Discussion

In this study, we describe otolaryngology residency PDs’ views about having their residents teach in medical school gross anatomy labs. We found that PDs whose residents were already teaching tended to view this process positively, and it was considered beneficial to the residents, medical students, and the residency program itself. The majority of Non-Teaching Program PDs endorse residents teaching but report not being asked to provide residents as the main factor for not participating. Therefore, it appears that the majority of otolaryngology PDs view residents teaching positively.

It is worthwhile to note again that medical students report increased comprehension of material after being taught by residents. Glasgow et al. found that more than 95% of first-year medical students reported an increased understanding of abdominal anatomy after being taught by surgical residents [3]. Another study found that the majority of students felt they had a better understanding of skin examination and lesions after attending an anatomy laboratory session where three dermatology residents and one attending physician were present to answer questions [4]. A third study performed by Shah et al. administered pre- and post-session anatomy quizzes to first-year students before and after multiple sessions with radiology residents reviewing chest, abdomen and pelvis, head and neck, and extremity anatomy. Their results showed that the students’ anatomy knowledge increased significantly, with quiz scores increasing 29.9%. Five students have also reported a positive outlook on having residents as teachers, and they are equally as effective as faculty after undergoing the relevant training [11].

This system is an aid to the residents as well. It allows them to review the relevant anatomy and gives them more exposure to teaching. Shah et al. found that after teaching medical students in the first-year anatomy course, radiology residents felt they were more effective at being able to teach radiologic and CT anatomy [5]. McBride and Drake investigated a program that has residents prepare and present cadavers to first-year medical students. They collected opinions about the program from both the residents and residency program directors. Residents reported the experience reinforced their knowledge of anatomy, and they enjoyed the opportunity to interact with and teach students. Program directors also reported positive feelings towards the arrangement, and they were pleased to have a way for their residents to review anatomy and to have the role of a medical educator [8]. Fitzpatrick et al. (2001) performed a study in which surgical residents reviewed gross anatomy via laparoscopy on a cadaver with first-year medical students. They reported that this experience gave the residents opportunities to learn how to use laparoscopy and to practice the technique without the worry of time constraints or negative consequences if a mistake were to occur [1].

In addition, allowing residents to teach can also be beneficial to the residency program itself. A previous study found that in a group of U.S. medical students applying to otolaryngology residency programs, 78% indicated their main influence toward applying to this field was their interaction with otolaryngology residents [12]. This supports the claim that allowing residents to teach junior medical students can aid in recruitment to the specialty.

Non-Teaching Programs may want to consider redesigning their resident curriculum in such a way as to protect time for residents to interact with and teach junior medical students. If the Non-Teaching Programs were not asked to provide residents by the medical school leadership, it may be worthwhile for them to consider offering this service, either as part of the established curriculum or as part of medical student extracurricular activities, such as ear, nose, throat (ENT) Interest Groups and extracurricular workshops. Spiers et al. described a one-day otolaryngology workshop run for volunteer medical students in the UK, where they demonstrated a statistically significant increase in positive perceptions of the specialty and interest in a career in otolaryngology [13]. This is an example of the kind of creative thinking educators in our specialty need to do to address the dearth of the otolaryngology curriculum in current medical schools. Davies and Elhassan showed that in UK medical schools, clinical otolaryngology comprised 1% of the undergraduate curriculum [14] while 75% of physicians working in UK emergency departments [15] and general practices felt that their undergraduate otolaryngology training was inadequate [16].

Limitations 

The response rate was low but similar to previous studies of this group of PDs [17]. Our study looked only at PD attitudes, so the point of view of residents, students, and medical school administrators is not described. In addition, our study did not assess why PDs felt they did not have enough residents or why the residents were unable to leave clinical duties. This may be an area for potential research, as teaching is a prominent duty of senior residents and attending physicians and should be integrated into residency curriculums. Lastly, there is the possibility of biases in the survey or the respondents, and because each program is different, the findings may not be able to be generalizable to an individual program.

## Conclusions

Our group of otolaryngology PDs felt that having residents teach undergraduate medical students is a positive addition to their programs. The literature shows that medical students and residents derive benefit from this near-peer teaching. Our results suggest that otolaryngology departments are not being invited to teach in many cases. Otolaryngology PDs may want to structure their programs in such a way that they are able to offer resident teachers, particularly in the medical students' anatomy lab, or consider accessing medical students via extracurricular activities.
